# Reconfiguration of Brain Network Dynamics in Autism Spectrum Disorder Based on Hidden Markov Model

**DOI:** 10.3389/fnhum.2022.774921

**Published:** 2022-02-08

**Authors:** Pingting Lin, Shiyi Zang, Yi Bai, Haixian Wang

**Affiliations:** ^1^School of Biological Science and Medical Engineering, Southeast University, Nanjing, China; ^2^Key Laboratory of Child Development and Learning Science (Southeast University), Ministry of Education, Nanjing, China; ^3^Research Center for Learning Science, Southeast University, Nanjing, China

**Keywords:** autism spectrum disorder, Hidden Markov Models, large-scale whole-brain network, global temporal dynamics, modularity analysis

## Abstract

Autism spectrum disorder (ASD) is a group of complex neurodevelopment disorders characterized by altered brain connectivity. However, the majority of neuroimaging studies for ASD focus on the static pattern of brain function and largely neglect brain activity dynamics, which might provide deeper insight into the underlying mechanism of brain functions for ASD. Therefore, we proposed a framework with Hidden Markov Model (HMM) analysis for resting-state functional MRI (fMRI) from a large multicenter dataset of 507 male subjects. Specifically, the 507 subjects included 209 subjects with ASD and 298 well-matched health controls across 14 sites from the Autism Brain Imaging Data Exchange (ABIDE). Based on the HMM, we can identify the recurring brain function networks over time across ASD and healthy controls (HCs). Then we assessed the dynamical configuration of the whole-brain networks and further analyzed the community structure of transitions across the brain states. Based on the 19 HMM states, we found that the global temporal statistics of the specific HMM states (including fractional occupancies and lifetimes) were significantly altered in ASD compared to HCs. These specific HMM states were characterized by the activation pattern of default mode network (DMN), sensory processing networks [including visual network, auditory network, and sensory and motor network (SMN)]. Meanwhile, we also find that the specific modules of transitions between states were closely related to ASD. Our findings indicate the temporal reconfiguration of the brain network in ASD and provide novel insights into the dynamics of the whole-brain networks for ASD.

## Introduction

Autism spectrum disorders (ASD) are a group of complex neurodevelopment disorders, which are characterized by repetitive and characteristic patterns of behavior and difficulties with social communication and interaction (American Psychiatric Association, 2013; Guo et al., 2020). The current ASD prevalence in the general population is estimated to be approximately 1% or higher (Developmental Disabilities, Monitoring Network, Surveillance Year 2010 Principal Investigators, and Centers for Disease Control and Prevention, 2014). Previous neuroimaging studies have indicated that ASD is related to the anomalous responses in certain brain areas, significant alteration of functional, or structural brain network and disturbed neural synchronization between brain areas (Belmonte, 2004; Tang et al., 2014; Zhan et al., 2014; Cerliani et al., 2015; Duan et al., 2017). Especially, the majority of functional neuroimaging studies based on resting-state fMRI have shown the under-connectivity hypothesis has been proposed based on the reduced intraregional functional connectivity (FC) between default mode network (DMN) and sensory processing network (Belmonte, 2004; Just, 2004; Just et al., 2006, 2012; Anderson et al., 2010; Kana et al., 2011). For example, the FC between DMN [including the medial prefrontal cortex (mPFC), posterior cingulate cortex (PCC), and precuneus], temporal pole, and pallidum was significantly reduced (Yerys et al., 2015). Other studies also found a similar reduced FC between mPFC and primary motor and sensory cortices (Jung et al., 2014), even temporoparietal junction, insula, and amygdala (Von dem Hagen et al., 2012). Some resting-state fMRI studies have found the phenomena of the over-connectivity or a mixture of under- and overconnectivity in ASD (Keown et al., 2013; Supekar et al., 2013; Cerliani et al., 2015). Although the underconnectivity patterns are not consistent, the FC from resting-state fMRI provides new insights into the underlying neurological mechanisms for ASD.

Numerous previous findings in ASD are based on the assumption that resting-state FC is relatively stable over time, which neglects the spontaneous fluctuations in brain activity and dynamics of brain networks (Chen et al., 2017). However, abundant evidence has indicated that the human brain can be seen as a complex dynamic system, in which transition smoothly and continuously between brain states is directly related to cognitive function (Sheline et al., 2010; Deco et al., 2011; Gu et al., 2017; Lin et al., 2017). Recent works also suggest that the brain activity and brain network from the rest-stating fMRI are dynamic and that the dynamical FC states can be captured from resting-state fMRI (Allen et al., 2012; Hutchison et al., 2013b; Damaraju et al., 2014; Zalesky et al., 2014; Liu, 2016). Importantly, the latest studies about dynamic FC have shown that the specifically dynamic FC states found can be characterized by weaker connectivity strength in ASD and that state-related dynamic FC can improve the sensitivity in classifying ASD from the healthy (Wee et al., 2016; He et al., 2018; Xiaonan Guo et al., 2020). Therefore, advances from extensive studies have suggested that the dynamic FC may be a potential biomarker of ASD.

The most common technique used for dynamic FC is the use of sliding windows for resting-states fMRI (Wendling et al., 2009; Allen et al., 2014; Vidaurre et al., 2018a; Mokhtari et al., 2019). However, the sliding windows approach has some limitations (Hutchison et al., 2013a; Leonardi and van de Ville, 2015; Zalesky and Breakspear, 2015; Hindriks et al., 2016; Preti et al., 2017). For example, the neural process observed from brain activity is highly dependent on the window (e.g., the temporal width of the window and length of step). Fortunately, Hidden Markov Model (HMM) describes the brain activity as a dynamics sequence of discrete brain states in the timescales directly assessed from data, which can dramatically overcome the limitations of the previous sliding window approach (Vidaurre et al., 2016, 2017; Stevner et al., 2019). Previous studies have confirmed that the HMM can be able to capture dynamics of brain activity in minimal timescales (Quinn et al., 2018; Vidaurre et al., 2018b,^2019^; Stevner et al., 2019). For example, recent studies on magnetoencephalography (MEG) have found that the HMM can capture the dynamics of brain activities in a resting state as little as 100 ms (Quinn et al., 2018; Vidaurre et al., 2018b,^2019^). In addition, previous studies have suggested that the HMM can provide a rich description for brain dynamics in the short timescales and can be applied to probe the reconfiguration of the whole-brain dynamics for psychiatric illnesses [e.g., major depression disorder (MDD)] (Wang et al., 2020). In this study, we employed an HMM analysis in a large multicenter data of ASD to identify the brain states from resting-state fMRI in minimum timescale. We aim to investigate the temporal dynamics and to characterize the spatiotemporal specificity of brain activity in short timescale for ASD.

## Materials and Methods

### Participants

Resting-state fMRI data from 507 subjects, including 209 ASD subjects and 298 HCs, from 14 sits in the ADIDE database were analyzed in this study.^1^ The inclusion and exclusion were adopted as same as the previous studies (Di Martino et al., 2014; Chen et al., 2017). Briefly, all subjects were male, from these sites, in which 75% of subjects had a full-scale IQ score (FIQ). Only subjects with FIQ within 2 SD + mean across all the Autism Brain Imaging Data Exchange (ABIDE) samples and with mean framewise displacement (FD) below 2 SD + mean were included. Moreover, all the subjects had complete anatomical images and functional imaging scans. It is important to note that we tried to build a well-matched dataset to control the effects across sites, which were consistent with the previous study (Chen et al., 2017). Specifically, we first applied a data-driven algorithm that maximized the *p*-values of the group difference of age, FIQ, and mean FD (using two-sample *t*-tests). Then, to exclude the influence of the site interaction effect, we also maximized the *p*-values of the interaction effects between sites and diagnostic groups of age, FIQ, and FD (using ANOVA). Details of the demographic information are shown in Table 1.

**TABLE 1:** Demographics of participants.

	ASD (*n* = 209)	HC (*n* = 298)	Group comparisons (*p*-value)
Age	16.5 ± 6.2	16.8 ± 6.2	0.5642^a^
Site × group interaction	–	–	0.9642^b^
Full Scale IQ	111 ± 13	110 ± 11	0.7191^a^
Site × group interaction	–	–	0.8502^b^
Mean FD	0.14 ± 0.1	0.14 ± 0.1	0.7219^a^
Site × group interaction	–	–	0.8341^b^
High head motion timepoints^c^	14.99 ± 20.78	14.77 ± 21.26	0.9091^a^
**ADI-R**			
Social score	20 ± 5	–	–
Communication score	16 ± 4	–	–
RRB score	6 ± 3	–	–
**ADOS**			
Total score	12 ± 4	–	–
Social score	8 ± 3	–	–
Communication score	4 ± 1	–	–
RRB score	2 ± 1	–	–

*ADI-R, autism diagnostic interview-revised; ADOS, autism diagnostic observation Schedule; RRB, restricted and repetitive behaviors.*

*^a^The two-sample t-test.*

*^b^ANOVA.*

*^c^Defined as the number of the timepoints whose framewise displacement (FD) > 0.5 mm and the preceding time point and the following two timepoints.*

All the preprocessing of resting state-fMRI was performed using the Statistical Parameter Mapping 8 (SPM8)^2^ and the Data Processing Assistant for Resting-State fMRI (DPARSF) toolbox.^3^ The main preprocessing steps include removing the first 10 time-points, temporal and head motion correction, normalization to Montreal Neurological Institute (MNI), smoothing with a Gaussian kernel [full width at half maximum (FWHM) = 8 mm] and band-pass filtering (0.01–0.1 Hz). Spatial smoothing was a very common preprocessing step for functional brain images. However, there is a great deal of debate about the choice of smoothing kernel. Previous studies suggested the optimal FWHM was about 8 mm based on the influence of spatial smoothing on fMRI group activation and FC analysis. In addition, signals from white matter (WM) and cerebrospinal fluid (CSF), as well as 24 rigid body motion parameters, were regressed out. Finally, it was based on the automated anatomical labeling (AAL) template extract the averaged fMRI time series in 90 brain regions (including cortical and subcortical brain areas). The AAL template was one of the most frequently used in fMRI studies of FC for ASD. The previous study about non-rapid eye movement (NREM) sleep based on HMM analysis indicated that the results were highly robust across different templates (Stevner et al., 2019). This study was supported by the Academic Committee of the School of Biological Sciences and Medical Engineering, Southeast University, China.

### Hidden Markov Models

**FIGURE 1:**
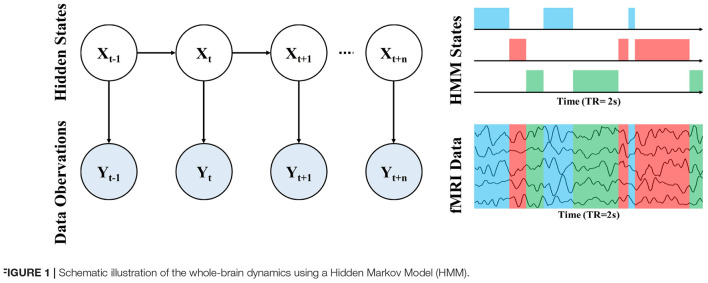
Schematic illustration of the whole-brain dynamics using a Hidden Markov Model (HMM).

To probe the alteration of whole-brain dynamics for ASD, we applied the HMM to obtain a group estimation of brain microstates. Briefly, the HMM can describe brain activity as a dynamic sequence of discrete recurring brain states (Vidaurre et al., 2016). All states are mutually exclusive and Markovian has the same probabilistic distributions but each has different distribution parameters. Thus, the states correspond to unique patterns of brain activity that recur in different parts of the time series. For each time point, a state variable dictates the probability of each state being active at that moment, thus we can determine which state is activated according to the maximum probability of each state. In other words, only one state may occur at a given time point and the next state is only dependent on the current state. Notably, we cannot directly observe the state from data because the state is abstract (hidden). The relationship between hidden states and our observed data can be observed through an observation model, as shown in Figure 1. In the HMM approach, each state has its observation model, from which we can obtain the probability distribution of our system is in that state based on the observed data.

In this study, we first obtained the representative time courses of 90 regions of interests (ROIs) for all subjects (including 209 patients with ASD and 298 HCs) though averaged all time courses over voxels within ROI. Then, 90-time courses from ROIs were demeaned by being divided by their standard deviation. Next, we respectively concatenate all time courses for each ROI across participants (including ASD and controls) and yield a data matrix. It is important to note that although it is possible to apply the HMM on each subject or each group independently, we applied the HMM on all the concatenated all-time courses. Thus, the states and the transition probabilities inferred by HMM were defined at the group level to obtain the matched states across ASD and HC. Finally, we estimated the number of recurring discrete states across through an HMM with multivariate Gaussian distribution. Importantly, each HMM state can be modeled as a multivariate normal distribution (including the mean activation distribution and an FC matrix). The mean activation distribution represents the mean level of state activity in brain regions. The FC matrix summarizes the pairwise temporal co-variations occurring between brain regions. Moreover, although the HMM was also considered as a tool for dimensionality reduction of data, there exist severe overfitting problems during estimating parameters per state due to the high spatial dimensionality of fMRI (Vidaurre et al., 2016). To address this problem, we carry out the principal component analysis for 90 ROI time courses across all the subjects before HMM inference as the previous studies (Vidaurre et al., 2016). We finally used the top 32 principal components, which keep approximately 90% of the signal variance.

### Choice of Number of Hidden Markov Model States

Based on the variational Bayesian inference, the HMM was implemented by probabilistically estimating the state statistics and transition probabilities (Vidaurre et al., 2016). Notably, there is a central and free parameter, the number of HMM states should be determined before HMM inference (Vidaurre et al., 2016; Stevner et al., 2019) because different numbers of states in practice offer only different levels of detail of brain dynamics. Previous studies have found that we can choose the number of states underlying the spontaneous brain activity in several ways, such as using quantitative measures like free energy or using non-parametric approaches (Vidaurre et al., 2016, 2017, 2018a,b, 2019; Stevner et al., 2019). Therefore, in this study, we evaluated the summary statistic (including minimum free energy and medial fractional occupancy) for the different number of HMM states with a range of 4–45.

### Analysis of Temporal Characterizations of Hidden Markov Model States

Upon the inferred HMM, a time course of probabilities can be assessed through the HMM Bayesian inference. Each value represents the probability that a state is active at a time point. Then we can compute the global statistics reflecting the properties and dynamics of the HMM state from the probabilities courses (Vidaurre et al., 2017; Quinn et al., 2018). In this study, we obtained three global temporal characterizations of HMM states, including fractional occupancies, lifetimes, and interval times. Specifically, fractional occupancies of HMM states are the ratio of the activated HMM states across the all-time course. The lifetime is computed as the amount of time spent in a state before moving into a new state, which interval time is calculated as the amount of time between consecutive visits to a state (Vidaurre et al., 2017; Quinn et al., 2018). These characterizations are the most commonly effectively captured within-subject temporal dynamics.

### Analysis of Transitions of Hidden Markov Model State

Furthermore, to investigate the organization of the transition probabilities, which were explicitly modeled by the HMM, a network-based clustering technique (also called the community detection technique) was adopted to the matrix of transition probability between HMM states. The HMMs states into a certain community have more frequent transitions with others in the same community than in other communities. In this study, we adopted a modularity maximization approach to detect the community of the matrix of transition probabilities and optimized the modularity quality function using Newman’s spectral community detection algorithm using Matlab function from the Brain Connectivity Toolbox^4^ (Newman, 2006; Rubinov and Sporns, 2010). Notably, we only keep 25% of the strongest transitions in a matrix of transition probabilities.

### Statistical Analysis

To test significant differences in global dynamics, we performed, respectively, two-tail two-sample *t*-tests for fractional occupancy, lifetimes, and interval times of HMM states between patients with subjects with ASD and HCs. In particular, age was considered an irrelevant variable and was regressed out to remove its effects. And the threshold *p* < 0.05 Bonferroni correction was set to determine the significance level. Noteworthy, although some measures during subject selection were adopted to avoid the effects of data sites or FIQ and a well-matched dataset was obtained in this study, we again performed, respectively, statistical analysis for temporal characterizations of HMM states with regressing out the sites and FIQ to verify the robustness of the results.

## Results

### Nineteen Hidden Markov Model States Were Identified Using Hidden Markov Model

**FIGURE 2:**
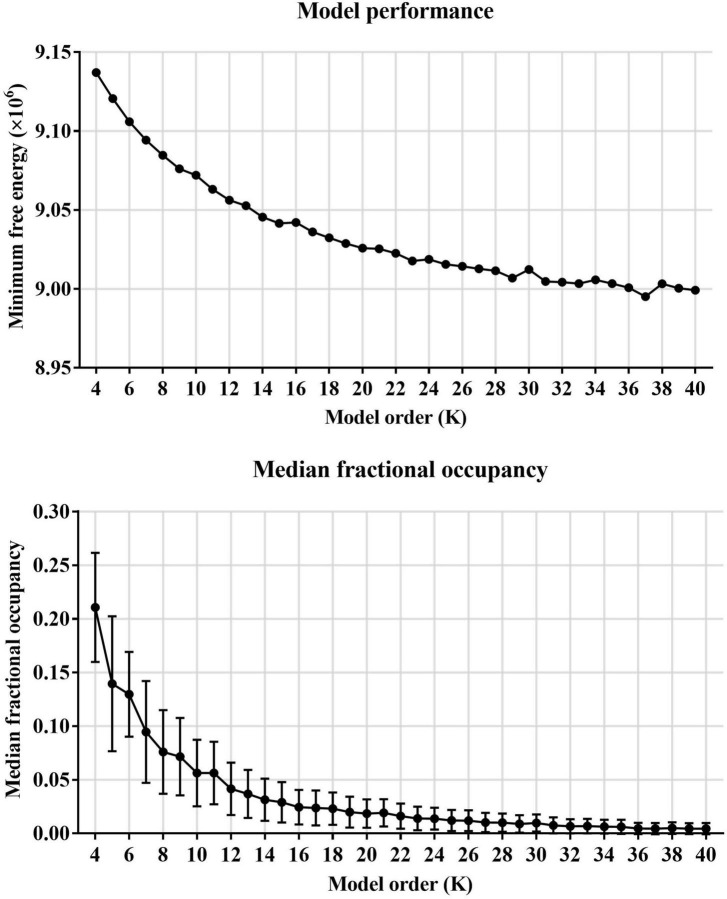
The choice of the number of HMM states.

First, HMM states were estimated on resting-state fMRI data from 517 subjects (including 209 subjects with ASD and 298 HCs). Before estimating HMM states, the minimum free energy, and medial fractional occupancy were used to determine the number of HMM states. The global statistics mainly included minimum free energy and medial fractional occupancy across the HMM states. As shown in Figure 2, we found that the minimum free energy was monotonically decreased with the increase of the number of HMM states, showing no negative peak. Therefore, consistent with previous studies of HMM, the free energy failed to provide valid information for the choice of the number of HMM states. A similar phenomenon is also reflected in the development of median fractional occupancy. Fortunately, we found that the median fractional occupancy decreased rapidly for the smaller number of states and ceased around the number for 19, which suggested a higher number of states might cause the occurrence of sporadic states, which might only appear in a few subjects. Hence, Based on these results above, this study finally estimated 19 HMM states.

### Global Temporal Statistics Exhibited Specific Alteration for Autism Spectrum Disorder

**FIGURE 3:**
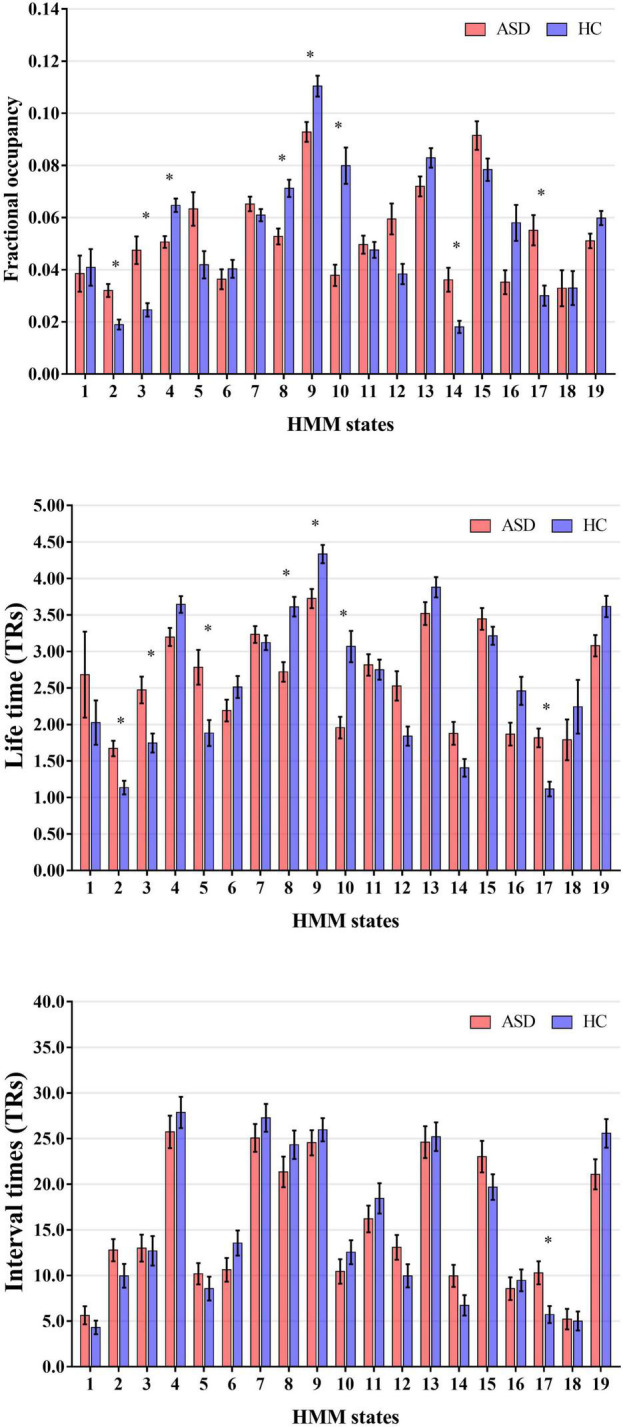
Alteration of the global temporal characterizes in autism spectrum disorder (ASD). *Represented the significant difference between ASD and HC (*p* < 0.05).

Then, to probe the temporal alterations of brain network for ASD, we calculated and compared the global temporal statistics of HMM states, including the fractional occupancies, lifetimes and interval times. Specifically, as shown in Figure 3, compared to HCs, we found that fractional occupancies of HMM states 4, 8, 9, and 10 for ASD subjects were significantly decreased (Without regressing out sites and FIQ: State 4: *p* = 4.71 × 10^–5^, *t*-value = −4.09; State 8: *p* = 4.78 × 10^–5^, *t*-value = −4.09; State 9: *p* = 0.0015, *t*-value = −3.18; State 10: *p* = 3.93 × 10^–7^, *t*-value = −5.12; With regressing out sites and FIQ: State 4: *p* = 1.00 × 10^–4^, *t*-value = −3.90; State 8: *p* = 5.7 × 10^–4^, *t*-value = −3.46; State 9: *p* = 4.5 × 10^–4^, *t*-value = −3.52; State 10: *p* = 2.11 × 10^–6^, *t*-value = −4.78; two-tailed two-sample, Bonferroni correction). And fractional occupancies 2, 3, 14, and 17 for ASD were significantly increased (Without regressing out sites and FIQ: State 2: *p* = 3.03 × 10^–5^, *t*-value = 4.20; State 3: *p* = 8.01 × 10^–5^, *t*-value = 3.96; State 14: *p* = 3.61 × 10-4, *t*-value = 3.58; and State 17: *p* = 3.07 × 10^–4^, *t*-value = 3.66; With regressing out sites and FIQ: State 2: *p* = 2.09 × 10^–4^, *t*-value = 3.73; State 3: *p* = −0.0015, *t*-value = 3.19; State 14: *p* = 2.04 × 10^–4^, *t*-value = 3.73; and State 17: *p* = 0.0049, *t*-value = 2.82; two-tailed two-sample, Bonferroni correction). As expected, lifetimes of participants with ASD had a similar alteration with fractional occupancies. Lifetimes of ASD subjects expressed significantly decreased in HMM 8, 9, and 10 (Without regressing out sites and FIQ: State 8: *p* = 1.24 × 10^–6^, *t*-value = −4.89; State 9: *p* = 0.0012, *t*-value = −3.26; State 10: *p* = 1.86 × 10^–6^, *t*-value = −4.31; With regressing out sites and FIQ: State 8: *p* = 5.11 × 10^–5^, *t*-value = −4.07; State 9: *p* = 0.0017, *t*-value = −3.15; State 10: *p* = 1.51 × 10^–4^, *t*-value = −3.81; two-tailed two-sample, Bonferroni correction) and increased in HMM 2, 3 and 17 (Without regressing out sites and FIQ: State 2: *p* = 2.76 × 10^–4^, *t*-value = 3.62; State 3: *p* = 0.0016, *t*-value = 3.23; State 17: *p* = 3.45 × 10^–5^, *t*-value = 4.16; With regressing out sites and FIQ: State 2: *p* = 0.0026, *t*-value = 3.03; State 3: *p* = 0.006, *t*-value = 2.75; State 17: *p* = 0.001, *t*-value = 3.28; two-tailed two-sample, Bonferroni correction). Meanwhile, interval times of HMM State 17 was increased for ASD subjects (Without regressing out sites and FIQ: State 17: *p* = 0.003, *t*-value = 4.17, two-tailed two-sample, Bonferroni correction). Our results indicated that there was the temporal reconfiguration of large-scale brain network for ASD subjects, which were able to be characterized by global temporal statistics of brain microstates.

### Specific Community Structure of Transitions for Autism Spectrum Disorder

**FIGURE 4:**
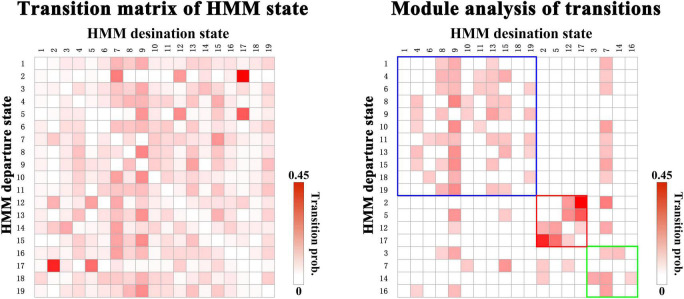
The modules of transitions between HMM states.

Next, we organize the whole-brain network states into a transition map and carried out a modularity analysis about the 19 × 19 transition probability matrix. Based on the most frequent transitions between the HMM states across all the subjects (including subjects with ASD and HCs), we found that the transition map was organized into a specific community structure with three partitions (modularity index = 0.3332) (Figure 4). Further combined with the global statistics, three modules in the transition map were respectively considered as the HC-related module and the ASD-related modules (including modules I and II). The HC-related module was characterized by the HMM state 4, 8, 9, and 10, whose global statistics, including fractional occupancies and lifetimes, were significantly increased in HCs. Correspondingly, the HMM states 2 and 17 were included in the ASD-related module I and the HMM states 3 and 14 in the ASD-related module II. These HMM states were higher fractional occupancies and longer lifetimes for subjects with ASD.

### Spatial Activation Maps of Whole-Brain States for Autism Spectrum Disorder

**FIGURE 5:**
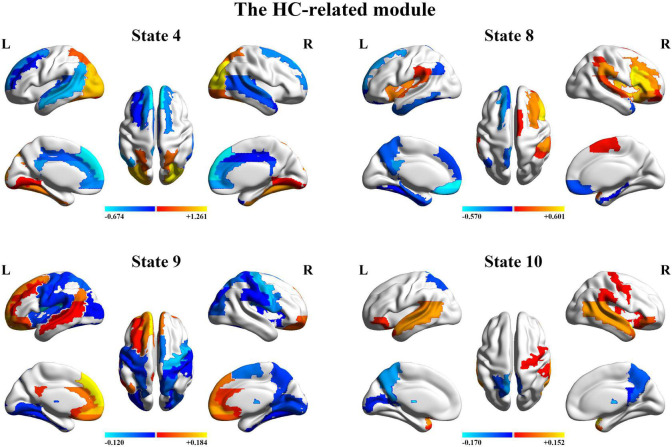
The mean activation maps for states 4, 8, 9, and 10 in the healthy control (HC)-related module.

**FIGURE 6:**
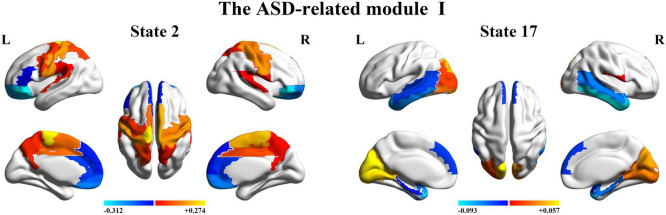
The mean activation maps for states 2 and 17 in the ASD-related module I.

**FIGURE 7:**
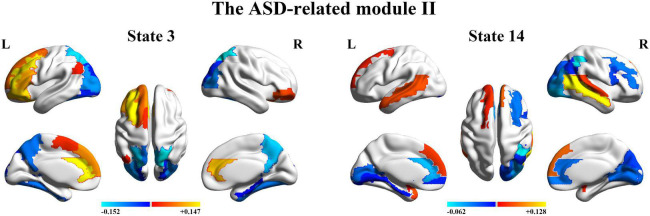
The mean activation maps for states 3 and 14 in the ASD-related module II.

Finally, we further probed the spatial activation maps of the whole-brain microstates in the HC-related module and the ASD-related module. As shown in Figure 5, we found that in the HC-related module, the HMM state 4 was mainly characterized by the decreased activation in the bilateral medial and superior frontal gyrus, the bilateral middle and superior temporal and the cingulate cortex [including the bilateral anterior and posterior cingulate cortex (ACC and PCC)] and the increased activation in the bilateral superior and middle occipital gyrus and fusiform. The HMM state 8 showed the decrease in the bilateral medial orbitofrontal gyrus, the left PCC, precuneus, para-hippocampal and angular, the bilateral rectus, and the bilateral medial temporal pole and the bilateral superior temporal gyrus, the bilateral inferior frontal gyrus, and the supramarginal gyrus. The HMM state 9 showed the decreased activation in subcortical areas and sensory (the bilateral thalamus, the bilateral putamen, left insula, and the bilateral pallidum) and motor and sensory areas (including the bilateral central gyrus, bilateral lingual gyrus, the bilateral Rolandic operculum area, the bilateral middle occipital gyrus, and the bilateral supramarginal gyrus), and the increased activation in DMN (including the bilateral medial and superior frontal gyrus, the bilateral anterior and PCC and the bilateral rectus). The HMM state 10 was mainly characterized by decreased activation in the bilateral thalamus, precuneus, PCC, calcarine lingual gyrus, and superior temporal gyrus and by increased activation in the bilateral superior and medial temporal and inferior orbitofrontal gyrus. Then we found the ASD-related module included two partitions. One was mainly characterized by the HMM state 2 and 17 (the ASD-related module I) and another by the HMM 3 and 14 (the ASD-related module II). As shown in Figure 6, in the ASD-related module I, the HMM state 2 showed the decrease in orbit-frontal gyrus, ACC, rectus, the superior and medial frontal gyrus, and the decrease in sensor and motor network and (including postcentral gyrus, paracentral lobule, supplementary motor area, and superior parietal lobule), auditory network (Heschel gyrus and superior temporal gyrus) and DMN (including PCC and precuneus). The HMM states 17 showed the decrease in temporal gyrus and superior and medial frontal gyrus and the increase in the visual and auditory network (calcarine, cuneus, lingual gyrus, and occipital gyrus). Meanwhile, as shown in Figure 7, in another ASD-related module (the ASD-related module II), state 3 showed the decrease in the visual network (including the bilateral occipital gyrus, fusiform and superior parietal gurus), and the increase in the DMN (the bilateral ACC and the bilateral superior and medial frontal gyrus). State 14 showed the decreased activation in the bilateral inferior parietal gyrus, the ACC, the middle frontal gyrus, and the middle and inferior occipital gyrus and the increased activation in the middle temporal gyrus.

## Discussion

In this study based on a large sample dataset, we probe the dynamic complexity and dissimilarity in spatiotemporal patterns of brain activity between subjects with ASD and the health. We identified 19 recurring brain states for all subjects from multicohorts through the HMM approach and found the temporal reconfiguration of HMM states for ASD, which provides a richer temporal description in a smaller time scale compared with previous studies using the sliding-window approach. Meanwhile, we adopted the community analysis to the HMM transition map and found the special transition pattern between HMM states for subjects with ASD, which previous studies did not fully capture.

Previous studies based on fMRI have identified dynamic re-configurations of large-scale brain activity voxel-wise changes (Guo et al., 2017), changes in connection strength (Anderson et al., 2010; Cerliani et al., 2015; Guo et al., 2018), or through long-temporal dependencies of the blood oxygen level dependent (BOLD) signal (Falahpour et al., 2016; Chen et al., 2017; He et al., 2018) for ASD. However, HMM analysis is a probabilistic representation in the space of whole-brain network states and transitions, which might provide novel insights into neural mechanisms of ASD. Here, 19 HMM states recurring across time were identified for ASD and the healthy, which was consistent with previous findings that FC of the brain is highly dynamic and can represent flexibility in functional coordination between distinct brain systems (Watanabe and Rees, 2017; He et al., 2018; Guo et al., 2020). Further, we analyzed the alteration of the global temporal characterization for each HMM state. Our results showed that compared to the health, fractional occupancies of states 4, 8, 9, and 10 were significantly decreased in ASD, while fractional occupancies of states 2, 3, 14, and 17 were significantly increased. These findings are consistent with previous findings that subjects with ASD and HCs have a significantly different pattern of state organization. Numerous previous studies have reported the significant alteration in time-vary patterns in ASD. For example, recent work observed that ASD significantly increased durations of functional connections in both individual brain regions and distributed networks, and the abnormal alteration was closely related to the disease severity (King et al., 2018). Further, the width of cross-correlation curves between resting-states fMRI time series could be used as a metric of the relative duration of synchronous activity between brain regions (also called “sustained connectivity”) in ASD (King and Anderson, 2018). In addition, the sustained connectivity in ASD may limit the ability to rapidly shift one brain state to another and is negatively related to processing speed and sustained connectivity (King and Anderson, 2018). Meanwhile, other studies also found that impaired cognition function in ASD may be associated with the alteration of brain states. Interestingly, the late study using an energy-landscape analysis reported that high-functioning adults with ASD show fewer neural transitions due to an unstable intermediate state (Watanabe and Rees, 2017). Similar aberrant temporal dynamics were reported by the study by Rashid et al. (2014) who found longer dwell times related to a globally disconnected state in youth with higher autistic traits. A mass of studies has found that individuals with ASD and health showed significantly different occurrences in two functional states. Particularly, individuals with ASD spent more time in a state with weak dynamical FC patterns and less negative dynamic FC between DMN and other networks, while the health spent more time in a state with both positive and negative dynamical FC patterns (Rabany et al., 2019). In summary, our results provide new insights about the dynamical alteration of brain activity in ASD and offer additional evidence for the evaluation of the impacts of psychiatric ASD.

To further, we combined with the community detection for the transition map between HMM states and indicate that ASD and HCs have respectively their specific community structures for the transition map between HMM states. Particularly, two modules are closely related to ASD. The ASD-related module I was characterized by the HMM states 2 and 17 and The ASD-related module II was characterized by the HMM states 3 and 14. Noteworthy, in ASD-related module II, two characterize HMM states showed the opposite trend of activation in the DMN. State 3 showed the increase in DMN (including ACC and superior and medial frontal gyrus), and state 14 showed the decrease in the corresponding area. Meanwhile, HMM states 3 and 14 showed similar activation in visual (including fusiform and parietal gyrus and occipital gyrus). In addition, we also found the other specific pattern of activation for the HMM states 2 and 17 in the ASD-related module I. Specifically, the HMM states 2 and 17 respectively showed a similarly increased activation in a part of sensory and motor network (SMN), visual, and auditory network. HMM state 2 showed the increased activation in SMN (including postcentral gyrus, paracentral lobule, supplementary motor area, and superior parietal lobule) and auditory network (including Heschel gyrus and superior temporal gyrus). The HMM state 17 showed increased activation in the visual and auditory network (calcarine, cuneus, lingual gyrus, and occipital gyrus). Our findings are also consistent with previous studies on ASD that suggest FC of DMN, SMN, visual, and auditory networks in ASD is significantly altered compared with controls. Previous studies have reported the decreased FC between ACC/mPFC and other DMN regions for individuals with ASD (Assaf et al., 2010; Weng et al., 2010). The decreased intra-hemispheric FC in mPFC, fusiform gyrus, and inferior temporal gyrus for ASD was also found (Anderson et al., 2010; Lee et al., 2016). The recent work also suggested that abnormalities in the above regions can be identified as neurofunctional markers for social impairments of ASD (Patriquin et al., 2016). Together with our results, these findings consistently highlight the potential role of the neural circuits associated with DMN (including ACC and mPFC) and sensory processing networks (including fusiform gyrus and inferior temporal gyrus) in the pathophysiological mechanisms underlying ASD. Meanwhile, consistent with the latest dynamical FC findings (Guo et al., 2018, 2020), our findings complement our understanding of the functional organization for ASD from a dynamic perspective.

Compared to the sliding window approach, the HMM approach provides a more rich description of brain dynamics without any predefined timescale information, e.g., the width of the window and step. However, two main methodological limitations of this study should be considered: the short-range dependency between HMM states and the number of HMM states. The former is mainly caused by two assumptions of the HMM applied to brain activity data. One assumption of the HMM is the state, which is mutually exclusive (Vidaurre et al., 2016, 2018b). Another assumption in the HMM approach is that when a state at a certain time point is known, the next state will be predicted without information of time courses before this point. So, there is a short-range dependency between HMM state occurrences, which is inconsistent with the previous study that the brain exhibits the long-range dependency of HMM states. Therefore, the HMM has methodological limitations for precisely characterizing the brain states. However, recent studies indicate the certain type of long-range dependency of HMM in the form of metastates, even when these are not explicitly parameterized in the model (Vidaurre et al., 2017, 2018a,b; Stevner et al., 2019). In a word, the HMM does not infer the long-range dependency of HMM, but we can freely discover the long-range dependency inherent to the bran data through the inferred HMM state sequence. The latter is mainly caused by the choice of the number of HMM states, which is a free parameter, and is difficult to determine a correct number of states according to the recording brain activity. Meanwhile, neither the sliding-window approach nor the HMM approach has capable of decomposing the explicit number of intrinsic states of brain activity (Hutchison et al., 2013a; Hindriks et al., 2016; Vidaurre et al., 2017; Stevner et al., 2019). In this study, we identify 19 HMM states according to the global temporal statists, e.g., free energy and median fractional occupancy, which is not a generalizing number for different brain datasets. In addition, there was also a limitation regarding the data organization in this study. Before the HMM inference, the subject-specific sets of 90 ROI timecourses were demeaned, divided by their SD, and concatenated across all the subjects to obtain a group estimation about the state. Noteworthy, it is possible to apply the HMM on each subject or each group independently to explore the different spatial patterns across the ASD and HC groups, which will bring a new challenge of state matching across subjects or groups. Moreover, more amount of time points can infer more robust states during HMM inference.

In summary, we propose a data-driven analysis approach (the HMM with multivariable Gaussian distribution) allowing the investigation of the dynamic alterations in the whole-brain network between ASD and controls. Based on the brain microstates identified by HMM, we found that the reorganization of intrinsic brain states at time scales for ASD and the special communities for ASD was characterized by the decreased activation in sensory processing networks (including visual network, auditory network, and SMN) and the increased activation in the DMN. These findings provide new insights into the large-scale dynamic circuit organization of the brain and suggest that brain dynamics should remain a prime target for further ASD research, especially regarding the intrinsic states underlying brain activity.

## Data Availability Statement

Publicly available datasets were analyzed in this study. This data can be found here: http://fcon_1000.projects.nitrc.org/indi/abide/.

## Ethics Statement

The studies involving human participants were reviewed and approved by the Academic Committee of the School of Biological Sciences and Medical Engineering, Southeast University, China. The patients/participants provided their written informed consent to participate in this study.

## Author Contributions

PL and SZ performed the statistical analysis and wrote the first draft of the manuscript. YB and HW contributed to the design of the study. All authors contributed to manuscript revision, read, and approved the submitted version.

## Conflict of Interest

The authors declare that the research was conducted in the absence of any commercial or financial relationships that could be construed as a potential conflict of interest.

## Publisher’s Note

All claims expressed in this article are solely those of the authors and do not necessarily represent those of their affiliated organizations, or those of the publisher, the editors and the reviewers. Any product that may be evaluated in this article, or claim that may be made by its manufacturer, is not guaranteed or endorsed by the publisher.

## Footnotes

1
http://fcon_1000.projects.nitrc.org/indi/abide/

2
http://www.fil.ion.ucl.ac.uk/spm

3
http://rfmri.org/DPARSF

4
https://www.nitrc.org/projects/bct
